# Conductive Hydroxyethyl Cellulose/Soy Protein Isolate/Polyaniline Conduits for Enhancing Peripheral Nerve Regeneration via Electrical Stimulation

**DOI:** 10.3389/fbioe.2020.00709

**Published:** 2020-07-03

**Authors:** Ping Wu, Yanan Zhao, Feixiang Chen, Ao Xiao, Qiaoyue Du, Qi Dong, Meifang Ke, Xiao Liang, Qing Zhou, Yun Chen

**Affiliations:** ^1^Department of Biomedical Engineering, School of Basic Medical Sciences, Wuhan University, Wuhan, China; ^2^Hubei Province Key Laboratory of Allergy and Immune Related Diseases, School of Basic Medical Sciences, Wuhan University, Wuhan, China; ^3^Department of Interventional Radiology, The First Affiliated Hospital of Zhengzhou University, Zhengzhou, China; ^4^Department of Ultrasound Imaging, Renmin Hospital of Wuhan University, Wuhan, China; ^5^Hubei Engineering Center of Natural Polymers-Based Medical Materials, Wuhan University, Wuhan, China

**Keywords:** hydroxyethyl cellulose, soy protein isolate, polyaniline, peripheral nerve injury, electrical stimulation

## Abstract

Nerve regeneration remains a challenge to the treatment of peripheral nerve injury. Electrical stimulation (ES) is an assistant treatment to enhance recovery from peripheral nerve injury. A conductive nerve guide conduit was prepared from hydroxyethyl cellulose (HEC)/soy protein isolate (SPI)/PANI sponge (HSPS) and then the HSPS conduits were used to repair 10 mm sciatic nerve injury in rat model with or without ES, using HSPS+brain-derived neurotrophic factor (BDNF) and autografts as controls. The nerve repairing capacities were evaluated by animal experiments of behavioristics, electrophysiology, toluidine blue staining, and transmission electron microscopy (TEM) in the regenerated nerves. The results revealed that the nerve regeneration efficiency of HSPS conduits with ES (HSPS+ES) group was the best among the conduit groups but slightly lower than that of autografts group. HSPS+ES group even exhibited notably increased in the BDNF expression of regenerated nerve tissues, which was also confirmed through *in vitro* experiments that exogenous BDNF could promote Schwann cells proliferation and MBP protein expression. As a result, this work provided a strategy to repair nerve defect using conductive HSPS as nerve guide conduit and using ES as an extrinsic physical cue to promote the expression of endogenous BDNF.

## Introduction

In clinical practice, electrical stimulation (ES) has been used to treat many diseases (Gunter et al., 2019). In the treatment of epilepsy, ES can reduce the frequency of seizures by increasing the seizure threshold (Charthad et al., 2018). In patients with myasthenia gravis, appropriate electrical muscle stimulation can promote recovery of muscle function (Gordon et al., 2010). ES can also be used to stimulate the heart after cardiac arrest. In the traditional Chinese acupuncture treatment, ES is also introduced to enhance the effect of acupuncture, which makes the modern improvement of traditional Chinese medicine (Chen et al., 2018; Fei et al., 2019). It suggests that ES has a great application prospect in clinical medicine.

In peripheral nerve injury, the treatment of ES has been getting more and more attention. Huang et al. (2012) demonstrated that an electrical environment with ES localized at the conductive scaffold is capable of accelerating nerve regeneration and promoting functional recovery in rats. Michael and coworkers reported that electrical muscle stimulation immediately following nerve transection enhances electrophysiological and behavioral recovery (Willand et al., 2014). Qi et al. (2013) highlighted the possibility of increasing the neurotrophins secretion in olfactory ensheathing cells (OECs) by combining the conductive scaffolds and ES. In addition, the current of ES was applicated to promote the neurite outgrowth *in vitro* (Durgam et al., 2010), and accelerate the recovery of facial nerve crush injury *in vivo* (Jang et al., 2018). Compared with the current of ES, the voltage of ES not only could promote neurite outgrowth, it also mediate the neuron and multiple stem cells differentiation without the addition of growth factors (Zhu et al., 2019), neuronal cell adhesion and viability (Arteshi et al., 2018; Hu et al., 2019), and motor function restoring (Gunter et al., 2019).

In recent years, it has reported that ES can enhance the expression of brain-derived neurotrophic factor (BDNF) by activating the Ca^2+^ and Erk signaling pathways (Wenjin et al., 2011). Specifically, levels of specific nerve growth factors or neurotrophic factors are downregulated in animal models of sciatic nerve injury, and the application of exogenous nerve growth factors or neurotrophic factors have a positive effect on the repair of nerve defects (Tajdaran et al., 2018; Liu et al., 2020). It is a common method to directly introduce exogenous neurotrophic factors into tissue engineered-nerve guide conduits (Labroo et al., 2018). The common methods used to mobilize growth factor in scaffolds are adsorption, encapsulation, entrapment, and covalent binding, in which adsorption of growth factor on prefabricated scaffold is a convenient, straightforward method (Yu et al., 2018). However, these exogenous neurotrophic factors in the nerve conduits are easy to be lost or be inactive (Hobson et al., 2000). The effects of these two methods on nerve repair induced by the secretion of endogenous nutrient factors via ES and the directly binding of exogenous factors to nerve guide conduits have not been reported in detail (Carvalho et al., 2019).

Many literatures reported that exogenous addition of growth factor could not promote nerve regeneration well, and growth factors were often limited by their high cost and the short half-life (Pan et al., 2013). For this reason, it is of great significance to activate and promote the secretion of endogenous neurotrophic factors instead of using exogenous neurotrophic factors. With the development of life science, many genetic modification techniques have been applied to tissue engineering, but they often raise the risk of tumorigenesis (Lu et al., 2013; Fatemeh et al., 2017). However, ES, a relatively safe method, gradually attracts people’s attention and is proved to promote the secretion of BDNF. BDNF is a polypeptide secreted by neurons, glial cells, and their innervating tissues (Xiao et al., 2009). BDNF maintains and promotes the development, differentiation, growth, and regeneration of various neurons (Lopes et al., 2017; Liu et al., 2020). It has been reported that the BDNF is synthesized in neurons and released from the surface of axon, the polarity of Schwann cells can be initiated by p75NTR which activated by BDNF, eventually led to the formation of myelin sheath by Schwann cells surrounding axon (Jonah et al., 2006). Therefore, BDNF plays an important role in the myelinization and the repair of nerve defects.

In order to achieve the goal of ES for nerve repair, the corresponding conductive conduits should be developed and used (Nezakati et al., 2018; Chen et al., 2019). In this work, we constructed a new functional composite conduit using conductive material and natural polymer materials. The basic nerve guide conduits are fabricated from hydroxyethyl cellulose (HEC) and soy protein isolate (SPI), the conductive material polyaniline (PANI) was *in situ* polymerization onto HEC/SPI conduits. And then the HEC/SPI/polyaniline sponge (HSPS) conduits were used to repair 10 mm sciatic nerve injury in rat model with or without ES. The effects of the ES and conductive conduit on the endogenous BNDF express and nerve repair, as well as the effects of exogenous BDNF on the growth of Schwann cells were investigated. This work attempted to provide a new cheap and environmentally friendly conduit combining *in vivo* ES method for the field of nerve regeneration and preliminary reveal the mechanism of ES and HSPS conduits to promote nerve regeneration.

## Materials and Methods

### Materials

Hydroxyethyl cellulose (viscosity, 30,000 mPa) was supplied by Shandong Head Reagent Co., Ltd. (Shandong, China). SPI with weight-average molecular weight (Mw) of 2.05 × 10^5^ was supplied by DuPont Protein Technology (Luohe, China). HEC and SPI were vacuum-dried for 24 h at 60°C before use. Aniline (purity 99.98%), epichlorohydrin (ECH, analytical grade, liquid, 1.18 g/mL), ammonium persulfate (APS), hydrochloric acid (HCl), and acetic acid were supplied by Sinopharm Chemical Reagent Co., Ltd. (Shanghai, China). Other chemicals were of analytical grade agents and used without further treatment.

### Preparation of HEC/SPI/Polyaniline Conduits

The HEC/SPI (HEC: SPI = 30: 70, w: w) conduits were prepared as our laboratory previous work (Zhao et al., 2017). The aniline monomer was dissolved in 1 M HCl solution, and the operating concentration of aniline is 0.2 M. Then the HEC/SPI conduits were dipped in aniline/HCl mixture and stirred for 30 min. The same volume of APS solution (0.2 M) was dropped into the aniline/HCl solution to induce the aniline *in situ* polymerization reaction for 2 h, which is the best polymerization time for conductivity according to our previous work (Wu et al., 2019). Then the HEC/SPI/polyaniline sponge (HSPS) conduits were rinsed with running water overnight to remove the acid, salt, and unpolymerized aniline for the animal experiments. The morphology of the HSPS conduits was observed on a scanning electron microscope (SEM, VEGA3, TESCAN, Czechia) with 20 kV as the accelerating voltage.

### *In vitro* Cell Experiments

#### Schwann Cells Isolation and Culture

The sciatic nerve of six neonatal rats (1–3 days) was harvested according to our previous protocol (Luo et al., 2015). Then the nerves were washed three times by PBS and cut into pieces, digested with 0.04% collagenase II and 0.25% trypsin in the shaker for 40 min, and then terminated and mechanically dispersed the digested nerve tissue. The crude suspension was centrifuged at room temperature for 15 min at 1500 r/min, abandoned the upper solution, added complete DMEM to disperse the collected cells. The cell suspension was seeded on T25 cell culture flasks and incubated at 37°C with 5% CO_2_. In order to deplete rapidly proliferating fibroblasts, the non-adherent cells were put into new cell culture flasks to continue culturing after 30 min. Then the Schwann cells were treated with cytosine arabinoside for another 48 h to increase the purity of Schwann cells. The medium was exchanged every other day. Schwann cells were marked by S100 immunofluorescence staining. Schwann cells were fixed with 4% paraformaldehyde for 20 min and incubated with the anti-S100 antibodies (GB11359, Servicebio) overnight at 4°C, washed three times with PBS, and then incubated with secondary antibody [GB21303, Cy3 conjugated Goat Anti-rabbit IgG (H+L), 1:100] at 25°C for 60–120 min.

#### Evaluation of Proliferation of Schwann Cells by MTT Assay

Schwann cells suspension were seeded in 96-well cell culture plates with 1 × 10^3^ cells/well and cultured at 37°C with 5% CO_2_ for 24 h. And then the culture mediums were replaced with fresh complete mediums containing different concentrations of BDNF (0, 10, 50, 100, 250, and 500 ng/mL). After incubating for 1, 2, and 3 days, the Schwann cells were treated with 20 μL 3-(4, 5-dimethylthiazol-2-yl) 2, 5-diphenyltetrazolium bromide solution (MTT, 5 mg/mL) and incubated for another 4 h. Then the MTT was removed from the 96-well cell culture plates. 200 μL dimethyl sulfoxide (DMSO) was added into the plates to dissolve the formazan crystals at 37°C for 15 min. The absorbance values were exam at 490 nm wavelength on a multi-well microplate reader (Multiskanfc, Thermo Scientific).

#### Western Blot

The Schwann cells were co-cultured with BDNF for 3 days and then the cells were harvested for western blot analysis. And then the Schwann cells protein extracts were prepared by Total Protein Extraction Kit (BestBio, China). All the cell proteins were mixed with sodium dodecyl sulfate–polyacrylamide gel electrophoresis (SDS–PAGE) loading buffer (P0015, Beyotime) and heated at 100°C for 5 min. Protein samples (80 μg/well) were loaded on SDS–PAGE (10–12%) and electroplated onto polyvinylidene fluoride (PVDF) films (Millipore, United States). The PVDF films were blocked with 5% non-fat milk (BD, United States) for 60 min at room temperature and subsequently incubated overnight at 4°C with the primary antibodies: anti-PCNA antibody (GB11010, Servicebio), anti-MBP antibody (GB11226, Servicebio), and rabbit β-actin Rabbit antibody (GB11001, Servicebio). After incubating the PVDF films with horseradish peroxidase (HRP)-labeled secondary antibodies (GB23303, Servicebio) for 60 min at 25°C, the signal was collected by Image Studio Digits Ver 4.0. Density values were normalized to β-actin. The quantification of Western blot data was performed using Image-J software.

### *In vivo* Animal Experiments

#### Surgical Procedures

All animal experiments were performed according to the “Guidelines and Regulations for the use and care of Animals of the Review Board of Hubei Medical Laboratory Animal Center,” based on the Experimental Animal Management Ordinance (National Science and Technology Committee of the People’s Republic of China, 1998). The animal study was reviewed and approved by the Animal Care and Welfare Committee of the Wuhan University. Sprague–Dawley (SD) rats (180–220 g, female) supplied by the Beijing Vital River Laboratory Animal Technology Co., Ltd., and acclimatized in the animal care facility for 1 week prior to surgery. The rats were anesthetized by intraperitoneal administration of 10% urethane sodium at a dose of 1 mL per 100 g body weight. After the anesthesia, the hair on the lateral thigh of the rats was shaven and the skin was treated with 75% alcohol solution. The incision extended from the lateral femoral oblique and the muscle tissue was split. Then the sciatic nerve was exposed in visual field. The nerve was cut with a sterile blade and the distance of defects about 8.0–10.0 mm. The animal experiments were divided into five groups (10 rats in each group), including Autograft, HSPS, HSPS+BDNF, HSPS-ES, and HSPS+ES. The detail information of the groups is listed in Table 1. HSPS conduits adsorbed 50 ng/mL BDNF polypeptide solution were labeled as HSPS+BDNF conduits. HSPS conduits with electrodes attached to conduit’s two ends but without subsequent ES were labeled as HSPS-ES. HSPS conduits with electrodes at conduit’s two ends and subsequent with ES was labeled as HSPS+ES. After the nerve were sutured with the HSPS conduits containing two electrodes, 1 h ES (3 V) was administered every 2 days, for a total of seven times. The four kinds of conduits with 12 mm length were used to bridge the nerve gap by 8-0 nylon sutures, respectively. The control group was the Autograft, in which the severed nerve stump was rotated 180° to bridge the nerve defect. Muscle and skin were closed with interrupted absorbable sutures. Post-operative animals were conventional breeding.

**TABLE 1:** Animal groups and HSPS conduits codes.

Group	HSPS conduit (12 mm)	BDNF	Electrodes	ES (3V)
HSPS	+	−	−	−
HSPS+BDNF	+	50 ng/mL	−	−
HSPS-ES	+	−	+	−
HSPS+ES	+	−	+	+
Autograft	−	−	−	−

*The “+” implies that the action element was included, and the “−” implies that the action element is not included.*

#### Effects of ES on the Expression of Endogenous BDNF *in vivo*

**FIGURE 1:**
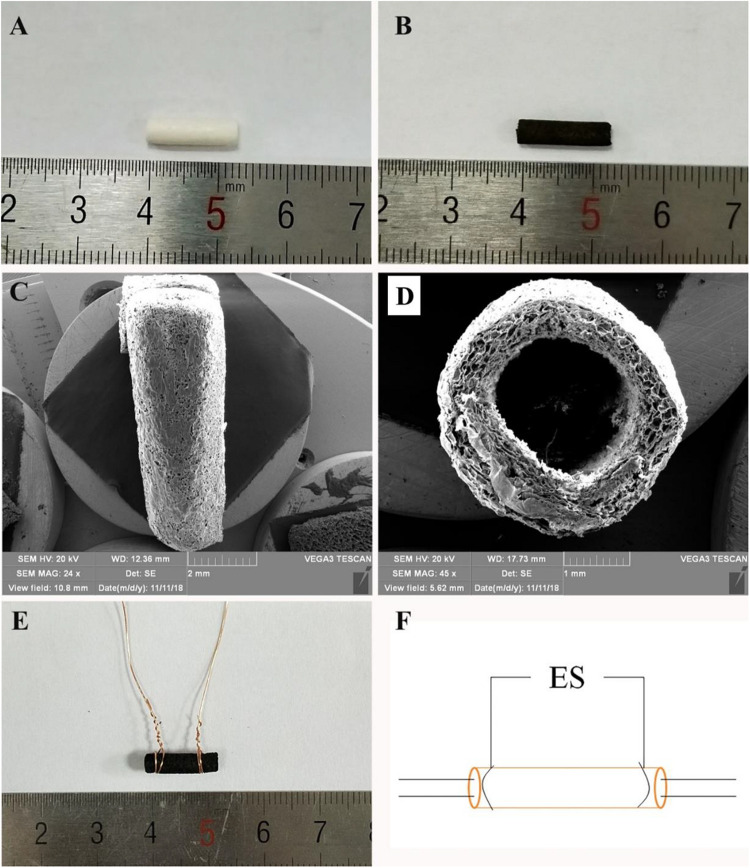
**(A)** General observation images of HEC/SPI conduit. **(B)** General observation images of HSPS conduit. **(C)** Longitudinal view SEM images of the HSPS conduit. **(D)** Transverse cross-section view SEM images of the HSPS conduit. **(E)** General observation images of HSPS-ES conduit with electrodes. **(F)** Schematic diagram of HSPS+ES electrical stimulation in animals.

To investigate the effect of ES on endogenous BDNF expression, BDNF immunofluorescence was detected. After the nerve was sutured with the HSPS conduits containing two electrodes (Figures 1E,F), ES was administered every 2 days for 60 min. After 2 weeks’ ES, the proximal nerve tissues from HSPS-ES and HSPS+ES groups’ rats (with and without ES) were fixed with 4% paraformaldehyde, paraffin-embedded to prepare immunofluorescence staining sections. The paraffin sections were deparaffinized and subjected to a heat-mediated antigen retrieval step using citrate buffer (pH 6.0). Then the samples were washed with PBS for three times, blocked with 5% BSA for 60 min, and incubated with the anti-BDNF primary antibody (A1307, Abclonal) at 4°C overnight. After washing with PBS for three times, the nerve tissue sections were incubated with HRP-labeled secondary antibodies (GB21303, Servicebio) for 60 min, and the cell nuclei were counterstained with DAPI. Finally, the immunofluorescence samples were observed by fluorescence microscope to evaluate the effects of ES to the BDNF endogenous secretion.

#### Evaluation of Motor Function

Walking track analysis was carried out for the assessment of never functional recovery. Three parameters were derived from the paw prints: print length (PL), toe spread (TS; distance from toe 1 to toe 5), and intermediate TS (IT; distance from toe 2 to toe 4). “N” and “E” represent the non-operated and operated hind limbs, respectively. The SFI value near to “−100” implies totally loss of function, while neared to “0” implies normal nerve function. The SFI was calculated with Eq. (1):

(1) SFI=-38.3×(EPL-NPL)/NPL+109.5×(ETS-NTS)/NTS+13.3×(EIT-NIT)/NIT-8.8.

#### Electrophysiological Examination

Three months after surgery, the sciatic nerve on the operated side was re-exposed under anesthesia. The electromyography was evaluated by an electrophysiology system (RM6240, China). The 10 mV electrical stimuli were applied to the nerve trunk at the proximal ends and the compound muscle action potentials (CMAPs) for the gastrocnemius belly on the ipsilateral side were recorded. Normal CMAPs were measured on the contralateral unoperated side.

#### H&E Staining

Three months after the operation, the regenerated nerve tissue or heart, liver, spleen, lung, and kidney tissue were harvested. The tissues were washed with normal saline and fixed in 4% paraformaldehyde solution for 24 h at 4°C. After tissues were dehydrated, paraffin, embedded, sectioned, and other steps, sections with thickness of 5 μm were obtained, and then H&E staining was performed. Finally, nerve or other tissue microstructures were observed under an optical microscope (BX51, OLYMPUS, Japan).

#### Myelin Analysis

At 3 months after surgery, the regenerated nerves that formed in place of conduits or autografts were quickly harvested after electrophysiological examination and fixed in cold 2.5% glutaraldehyde solution. The regenerated nerves were washed in PBS and sections were taken from the middle regions of the regenerated sciatic nerve. The samples were post-fixed with 1% osmium tetraoxide solution, dehydrated, and embedded in Epon 812 epoxy resin. Transverse semithin 1 μm sections were stained with toluidine blue and observed under a light microscope (BX51, OLYMPUS, Japan). The total area and the total number of myelinated nerve fibers were measured using Image-Pro Plus software. Transverse ultrathin 50 nm sections were stained with lead citrate and uranyl acetate, followed by examination under a transmission electron microscopy (TEM, HT7700, Hitachi, Japan). Image-Pro Plus 6.0 software was used to measure axon diameter and myelin sheath thickness from the TEM images.

### Statistical Analysis

All quantitative data were expressed as mean ± SEM. One-way ANOVA or *t*-test was used for statistics Analysis. The difference (*P* < 0.05) was considered to be statistically significant.

## Results

### Construction of HSPS Conduits

In aniline/HCl solution, yellow HEC/SPI conduits turn black after *in situ* polymerization of aniline induced by APS (Figures 1A,B). Figures 1C,D show the longitudinal section and cross-section of the HSPS conduit (Inner diameter = 1.8 mm, outer diameter = 2.0 mm, length = 12 mm) using SEM. The images show that the conduit also has a porous three-dimensional structure. In our previous work, the preparation method and conductive parameters of HSPS have been reported. The conductivity of HSPS was 1.7 S m^–1^, which was detected was by four-probe method. The mean resistance of the HSPS sponge was 20.6 kΩ detected in our previous work (Wu et al., 2019). Since both the sponge and the conduits are made from the same materials and used the same chemical reaction system, we can assume that the mean resistance of both the conduits and the sponge are 20.6 kΩ. In this research, the conductive HSPS was only prepared as conduit and applied with ES for peripheral nerve repair.

Figure 1E is the picture that the copper wires attached to both ends of the conduits, while Figure 1F is the schematic diagram of the *in vivo* ES. As shown in Figure 1F, the conductive HSPS and the electrodes form a circuit loop, and the external alternating current (3 V) is connected for ES.

### Effects of BDNF on Schwann Cell Proliferation and Myelination *in vitro*

**FIGURE 2:**
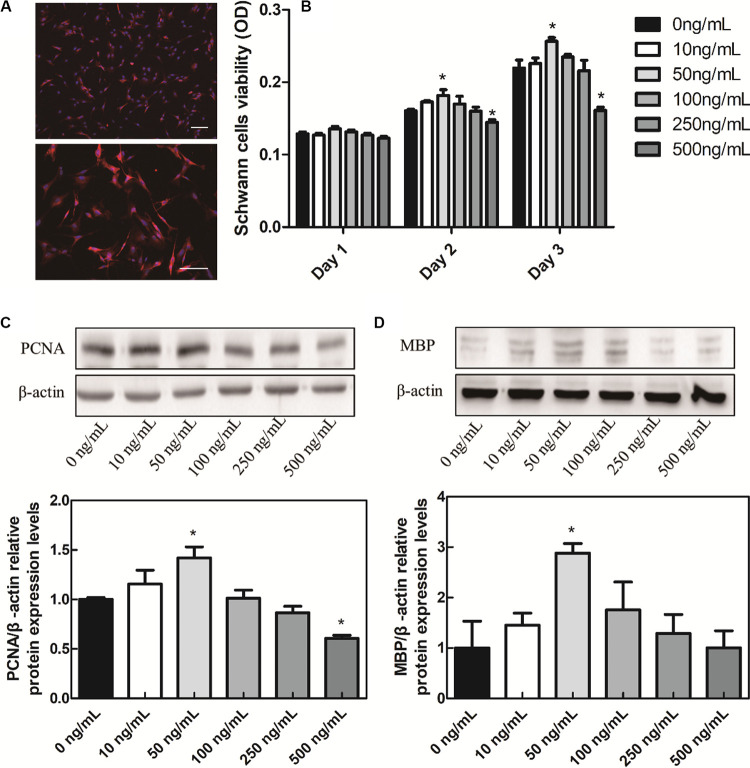
*In vitro* cell experiments. **(A)** Schwann cells S100 IF identification, scale bar = 100 μm. **(B)** BDNF promotes the Schwann cells proliferation. **(C)** BDNF promotes the Schwann cells PCNA. **(D)** MBP protein relative expression levels. *P < 0.05 compared with the 0 ng/mL group.

Many literatures have reported that the BDNF secretion can be promoted by ES and the polarity of Schwann cells can be initiated by p75NTR that activated by BDNF, which eventually led to the myelination by Schwann cells surrounding axon (Jonah et al., 2006). In order to study the BDNF mechanism on Schwann cell proliferation and myelination, the Schwann cells were extracted from 1–3 days neonatal rats. The purity of Schwann cells is always calculated by the ratio of S100 positive cell number to that of DAPI (Luo et al., 2015). As shown in Figure 2A, the purity of Schwann cells was over 95% in the present study. MTT experiments were used to verify the effect of BDNF on Schwann cell proliferation and results are shown in Figure 2B. On day 1, there were no significant differences among the groups. On day 2, 50 ng/mL BDNF group had a certain promotion effect on Schwann cells proliferation, while 500 ng/mL BDNF group had a certain inhibition effect. The MTT result of day 3 was consistent with that of day 2.

In order to verify the effects of BDNF on Schwann cell proliferation and myelination, western blot experiments were conducted. In eukaryotes, three DNA polymerases (alpha, delta, and epsilon) have been identified. DNA primase forms a permanent complex with DNA polymerase alpha. PCNA and RFC work as a clamp and a clamp loader. Therefore, in the process of Schwann cells proliferation, PCNA is a biomarker protein for cell proliferation, commonly used to identify cell proliferation (Yang et al., 2019). In addition, myelin basic proteins (MBPs) are a group of seven proteins produced from a single gene mapped to chromosome 18q22–q23 by alternate splicing, and found in the central and peripheral nervous system myelin. MBPs in Schwann cells are common participants in all myelinization, even in the most primitive vertebrates (Tuinstra et al., 2012). Different concentrations of BDNF were added into Schwann cells cultures to verify the effect of BDNF on the proliferation and myelinization of Schwann cells. We applied different concentrations of BDNF to Schwann cells, then detecting the PCNA and MBP expression. In the culture medium containing 50 ng/mL BDNF protein, the PCNA and MBP proteins expression in Schwann cells were significantly upregulated (Figures 2C,D). Hence, it can promote the Schwann cells proliferation and myelinization by controlling the exogenous addition of BDNF. In addition, many literatures reported that the BDNF secretion can be enhanced by ES (Wenjin et al., 2011). Therefore, either direct addition of BDNF or ES can improve Schwann cell proliferation and myelinization.

On the contrary, the PCNA protein decreased under the 500 ng/mL BDNF protein levels, so the appropriate BDNF protein concentration would promote Schwann cells proliferation while the excessive BDNF would inhibit their proliferation, which was consistent with the MTT results. In addition, adsorption of growth factor on prefabricated scaffold is a convenient and straightforward strategy to mobilize growth factors in tissue engineering scaffolds (Singh et al., 2012). It could circumvent exposure to harsh conditions, which is especially suitable for the HSPS scaffold fabrication. Therefore, HSPS conduits adsorbed 50 ng/mL BDNF polypeptide were used as the exogenous BDNF nerve conduits group (HSPS+BDNF group) in our follow-up study.

### *In vivo* Evaluation

#### Effects of ES on BDNF Expression *in vivo*

**FIGURE 3:**
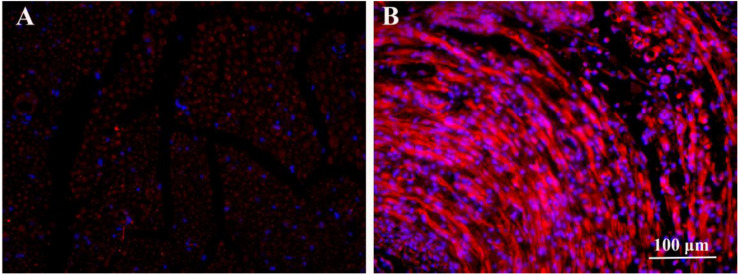
Immunofluorescence image of BDNF proteins in the nerve of HSPS-ES group **(A)** and HSPS+ES group **(B)**.

After 2 weeks of ES, the immunofluorescence images of BDNF secreted from the proximal injured nerve ends in HSPS-ES and HSPS+ES groups are shown in Figure 3. Rats in HSPS-ES group were not given ES, but the rats in HSPS+ES group received 3 V ES. Since the success of nerve regeneration is closely related to the secretion of early growth factors, we performed BDNF immunofluorescence analysis on the injured proximal nerve after 2 weeks of ES. The BDNF expression in HSPS+ES group is significantly higher than that of HSPS-ES group. This result is consistent with Huang’s result, the BDNF and NGF expression level would elevate under ES *in vivo* (Huang et al., 2010).

#### Sciatic Nerve Function Index (SFI) Evaluation

**FIGURE 4:**
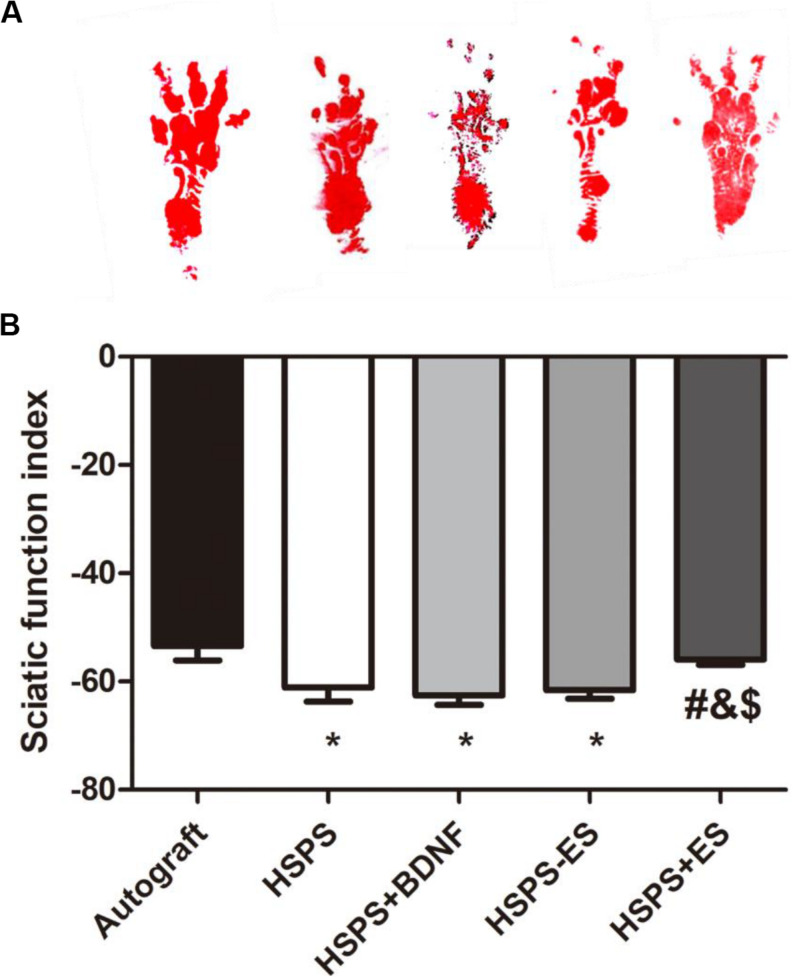
The sciatic functional index (SFI) in each group. **(A)** Images of footprints the SD rats and **(B)** statistical results of SFI values in each group at 3 months after surgery. *P < 0.05 compared with the Autograft group, ^#^*P* < 0.05 compared with the HSPS group, ^&^*P* < 0.05 compared with the HSPS+BDNF group, ^[*dollar*]^*P* < 0.05 compared with the HSPS-ES group.

The sciatic nerve function index (SFI) is used to evaluate the target muscle motor function of the sciatic nerve. At 3 months after surgery, the mean SFI values of Autograft, HSPS, HSPS+BDNF, HSPS-ES, and HSPS+ES groups were −53.5, −61.1, −62.5, −61.5, and −55.9, respectively. The mean SFI values of HSPS, HSPS+BDNF, and HSPS-ES groups were all lower than that of the Autograft and HSPS+ES group, while the mean SFI values of HSPS+ES and Autograft groups were similar (Figure 4). It can be seen that HSPS, HSPS+BDNF, and HSPS-ES conduits can support the recovery of sciatic nerve function of 10 mm nerve defects. Since two extra electrodes were needed for the ES, the HSPS-ES group was set, but the experimental results showed that the addition of the electrodes did not inhibit the function of the sciatic nerve. After the ES, the mean SFI value of the HSPS+ES group was higher than that of the other three conduit groups, so it illustrated that the effect of ES on the recovery of SFI was higher than that of the other conduits.

#### Electrophysiological Evaluation

**FIGURE 5:**
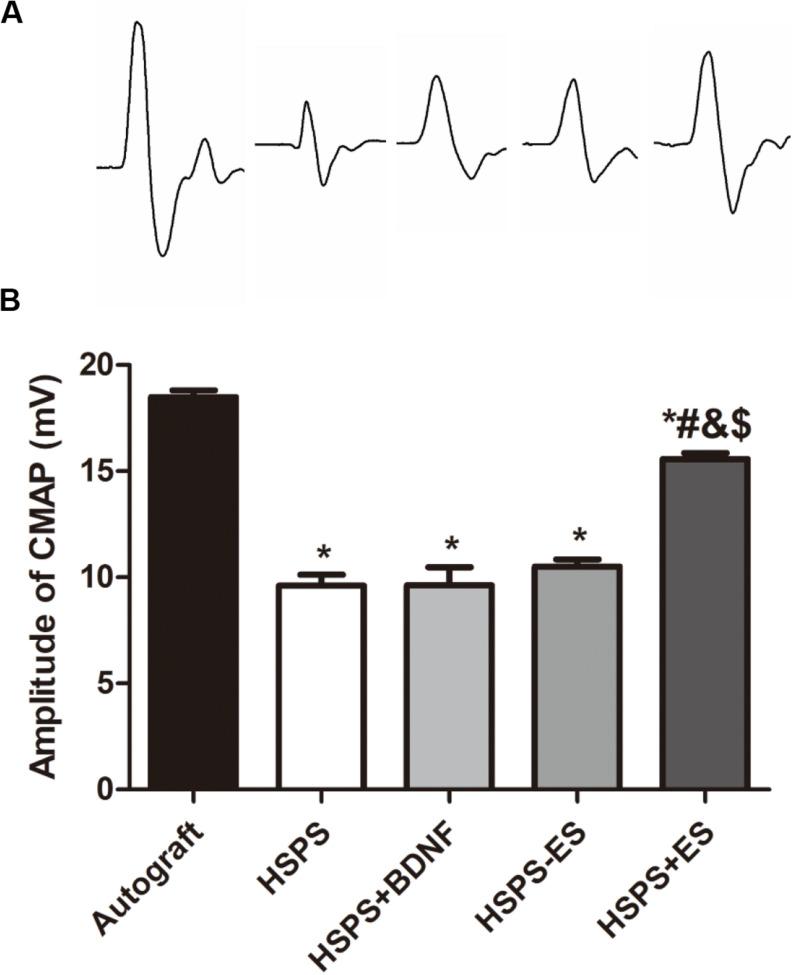
The results of electrophysiological test. **(A)** Representative CMAPs recordings on the injured side for each group. **(B)** The statistical results of peak amplitude of CMAPs. *P < 0.05 compared with the Autograft group, ^#^*P* < 0.05 compared with the HSPS group, ^&^*P* < 0.05 compared with the HSPS+BDNF group, ^[*dollar*]^*P* < 0.05 compared with the HSPS-ES group.

Three months after the operation, the peak amplitude of CMAPs was further recorded by the biological signal acquisition and analysis system. Figure 2C is representative CMAPs records of the surgical sides of each group. The CMAPs signals could be detected in each group, but the waveforms of signals were quite different among the five groups. The mean peak amplitudes of CMAPs in Autograft, HSPS, HSPS+BDNF, HSPS-ES, and HSPS+ES groups were 18.5, 9.6, 9.62, 10.5, and 15.6 mV, respectively (Figure 5). The mean peak amplitudes of CMAPs in HSPS+ES groups were significantly higher than those in HSPS, HSPS+BDNF, and HSPS-ES group, while the mean peak amplitudes of CMAPs in HSPS+ES groups were lower than that in Autograft group. The mean peak amplitudes of CMAPs in HSPS+ES group was significantly higher comparing with other conduits group, demonstrating that ES can promote the recovery of electrophysiological functions comparing with other conduit groups.

#### H&E Staining Analysis

**FIGURE 6:**
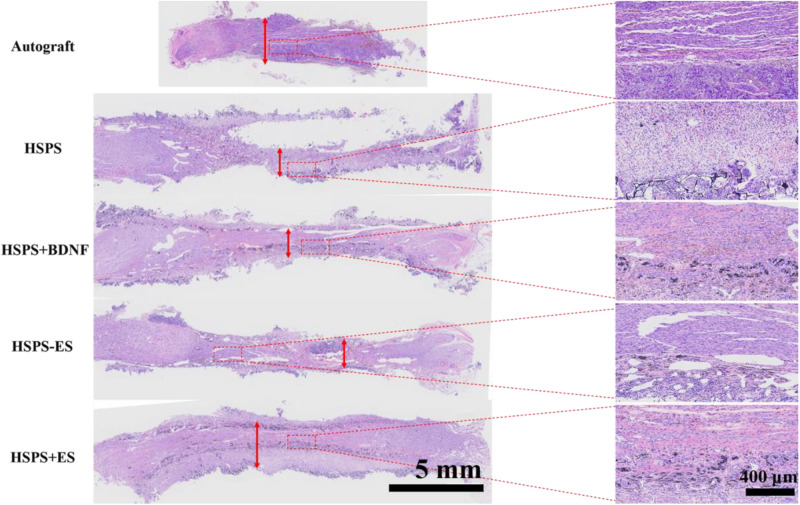
Images of HE-stained longitudinal sections of the regenerated nerve in each group at 3 months after surgery, the red arrows represented the diameter of the regenerated nerves.

Three months after the operation, H&E staining was performed on the longitudinal sections of regenerated complete nerves in the Autograft group, HSPS group, HSPS+BDNF group, HSPS-ES group, and HSPS+ES group, and the results are shown in Figure 6. After the 10 mm defect was truncated in the Autograft group, the 10 mm nerve truncated would shrink to 6–8 mm. The HSPS, HSPS+BDNF, HSPS-ES, and HSPS+ES group were all able to grow 10 mm nerve after 12 mm conduits were used to bridge. The mean diameters of regenerated nerve in Autograft, HSPS, HSPS+BDNF, HSPS-ES, and HSPS+ES groups were 1.485, 0.84, 0.807, 0.817, and 1.49 μm, respectively. The diameters of regenerated nerve in HSPS, HSPS+BDNF, and HSPS-ES groups were thinner than that in Autograft group and HSPS+ES group. The results of H&E staining showed that HSPS conduits combined with ES could increase the diameter of the regenerated nerve.

Moreover, in the enlarged illustration of HSPS, HSPS+BDNF, HSPS-ES, and HSPS+ES, it can be found that there are many black particles distributed in the regenerated nerve tissues. We speculate that these black particles are polyaniline particles from the degraded HSPS conduits.

#### Regenerated Myelin Analysis

**FIGURE 7:**
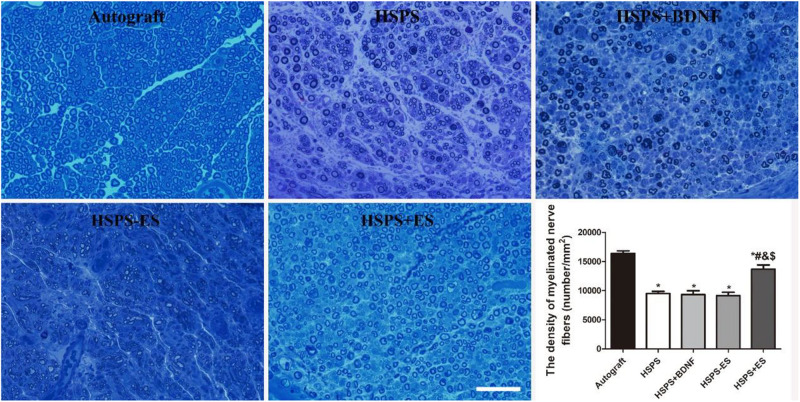
Images of toluidine blue stained regenerated nerve cross sections and the statistical density of myelinated nerve fibers in regenerated nerves for each group, scale bar = 20 μm. *P < 0.05 compared with the Autograft group, ^#^*P* < 0.05 compared with the HSPS group, ^&^*P* < 0.05 compared with the HSPS+BDNF group, ^[*dollar*]^*P* < 0.05 compared with the HSPS-ES group.

Toluidine blue staining of regenerated nerve tissues were investigated to evaluate myelin fiber density of different conduits. The results of toluidine blue staining on the cross-section of the middle segment of the regenerated nerve are illustrated in Figure 7. The Autograft, HSPS, HSPS+BDNF, HSPS-ES, and HSPS+ES groups all showed different sizes and densities of nerve fibers. The mean density of myelinated nerve fibers in Autograft, HSPS, HSPS+BDNF, HSPS-ES, and HSPS+ES groups were 1.64 × 10^4^, 0.94 × 10^4^, 0.93 × 10^4^, 0.91 × 10^4^, and 1.36 × 10^4^ number/mm^2^, respectively. The mean myelin fiber density of HSPS, HSPS+BDNF, and HSPS-ES groups was all lower than that of Autograft and HSPS+ES groups, while the mean myelin fiber density of HSPS+ES group was slightly lower than that of Autograft group.

**FIGURE 8:**
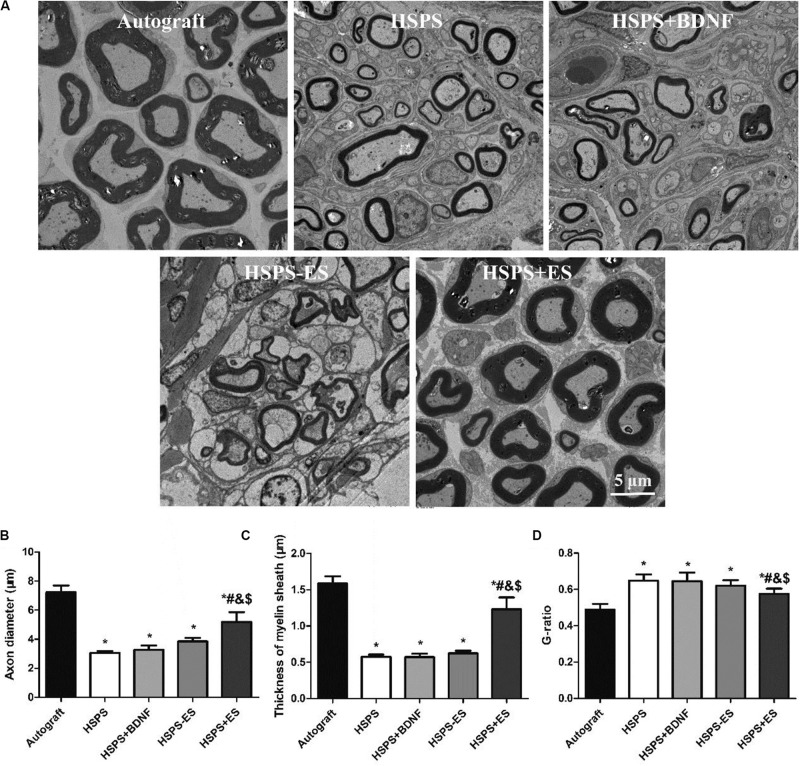
Images of toluidine blue stained regenerated nerve cross sections and the statistical density of myelinated nerve fibers in regenerated nerves for each group. **(A)** TEM images of the middle portion of regenerated nerve and statistical analysis of **(B)** axon diameter, **(C)** thickness of myelin sheath, and **(D)** G-ratio. *P < 0.05 compared with the Autograft group, ^#^*P* < 0.05 compared with the HSPS group, ^&^*P* < 0.05 compared with the HSPS+BDNF group, ^[*dollar*]^*P* < 0.05 compared with the HSPS-ES group.

Transmission electron microscopy observation of the middle segments of regenerated nerve tissues from each group is demonstrated in Figure 8. Different axon diameters and thicknesses of myelin sheaths are shown in Autograft, HSPS, HSPS+BDNF, HSPS-ES, and HSPS+ES groups. The axon diameters of HSPS, HSPS+BDNF, and HSPS-ES were smaller than that of Autograft and HSPS+ES, while the axon diameters of HSPS+ES group were lower than that of Autograft group. Second, the trend of myelin thickness is consistent with the trend of myelin diameter. Finally, the G-ratio is the ratio of the inner diameter of axon to the diameter of axon. The higher the G-ratio, the worse the effect of myelin repair effect. The G-ratio values of the three groups of HSPS, HSPS+BDNF, and HSPS-ES were greater than that of the Autograft and HSPS+ES groups, while G-ratio value of the Autograft group was lower than that of HSPS+ES group. After ES, the density of myelin fibers, axon diameters, and thicknesses of myelin sheaths in HSPS+ES group was higher than that in the other three conduit groups, so it can be said that ES can promote the remyelination. In addition, the density of myelin fibers, axon diameters, and thicknesses of myelin sheaths in the HSPS+ES group was slightly lower than that in the Autograft group, indicating that the remyelination could be increased to a large extent by electric stimulation, but the effect was not as high as that of Autograft group.

#### The Toxicity Assessment of the HSPS Conduits

**FIGURE 9:**
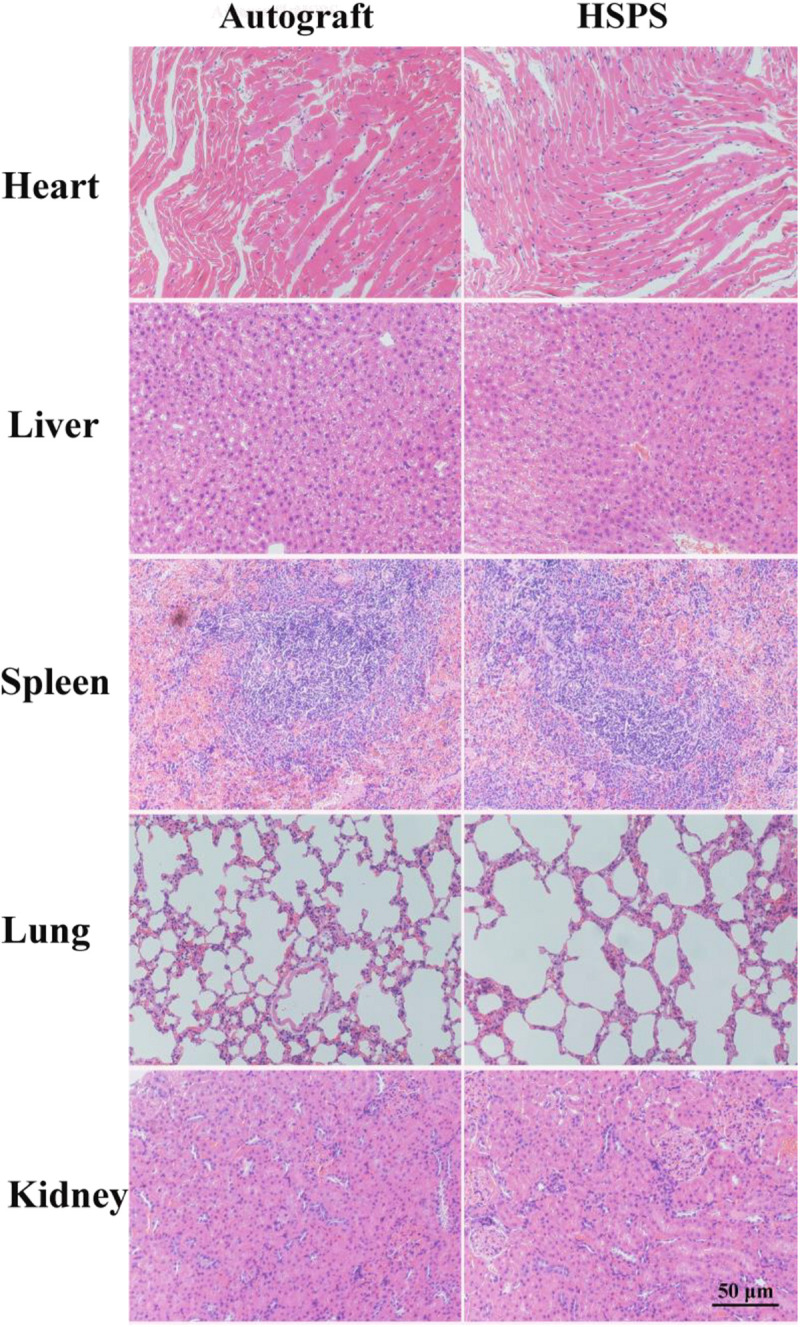
The toxicity assessment of the HSPS conduits *in vivo*.

In order to evaluate the toxic effect of polyaniline in HSPS conduits *in vivo*, H&E staining the heart, liver, spleen, lung, and kidney of rats in Autograft and HSPS conduit group 3 months after nerve repair surgery were harvested, sliced, and stained. Figure 9 shows that there was no obvious immune response in heart, liver, spleen, lung, and kidney tissues of rats in the Autograft group and the HSPS sponge nerve group, and the degraded polyaniline particles did not invade the organs, indicating that HSPS had no toxic effect *in vivo*.

## Discussion

In this study, an efficient strategy for peripheral nerve repair is developed by the combination of ES and conductive HSPS conduits. ES combining with conductive HSPS conduits can effectively promote the recovery of injured nerve motor function, electrophysiology, and morphology. All the indexes of nerve regeneration in HSPS+ES group were better than that of other conduits groups. In addition, the secretion of BDNF can be significantly promoted by ES *in vivo*. Many literatures have also proved that *in vitro* ES can promote the secretion of BDNF (Huang et al., 2012). In our *in vitro* cell experiments, it was also found that the appropriate concentration of BDNF can promote the proliferation and myelination of Schwann cells.

As stated by Du et al. (2018), the polycaprolactone nanofiber nerve conduits were used to repair the sciatic nerve defect in rats, but the ES did not play a positive role in the functional recovery of rats, which may be related to the non-conductive polycaprolactone conduits. When polypyrrole/chitosan conduits were applied to repair sciatic nerve defect in rats, ES group could promote the improvement of SFI value, peak amplitude of CMAPs, myelin fiber density, axon diameters, and thicknesses of myelin sheaths (Huang et al., 2012). In conclusion, ES on the basis of conductive conduit can promote the recovery of sciatic nerve function, while non-conductive conduits cannot support the corresponding recovery. There are several factors in this ES process. The first one is the electrode, the second one is the ES, and the third one is the conductivity of the conduits (Vijayavenkataraman et al., 2019). Our experiments showed that the electrode had no effect to nerve regeneration in ES. However, the literature has proven that conductive materials are necessary (Du et al., 2018). If non-conductive materials are used, the impact of ES is limited, because it can only directly act on the cells and cannot provide a large area of ES. According to the literature and our this work, it has been demonstrated that the improvement in SFI values, peak amplitude of CMAPs, myelin fiber density, axon diameters, and thicknesses of myelin sheaths are mainly due to the action of ES, which is carried out through the conductive conduits (Huang et al., 2012).

The SFI, electrophysiological, HE staining, myelin analysis results all illustrate that the exogenous BDNF could not significantly promote the nerve function recovery compared with the HSPS group. There are several possible reasons for exogenous BDNF failure of promoting nerve regeneration: The BDNF absorbed by HSPS conduits suffers from a short half-life due to its sensitivity to enzymes and chemical molecules *in vivo*; the sudden release of BDNF has inhibitory effects on nerve regeneration. Unlike exogenous BDNF groups, the continuous secretion of BDNF in HSPS+ES group was thought to protect motor and sensory neurons and stimulate the Schwann cells proliferation and myelination. Durgam et al. reported that PPy–PCL film significantly increased the number of PC12 cells bearing neurites compared to unstimulated PPy–PCL with ES. And the conductive PPy–PCL NGCs which were implanted in a 10 mm defect made in the sciatic nerve could support neurons proliferation and growth *in vivo* without ES (Durgam et al., 2010). Compared with Durgam’s work, we have set up a conductive nerve conduit plus ES group *in vivo* animal experiments, so we have demonstrated the effectiveness of ES *in vivo*. Jang et al. (2018) used ES to promote the recovery of facial nerve, but only the control group and the ES group were set up in the animal experiments. Comparing with Jang’s work, we not only set up the electrode group, but also set up the exogenous BDNF group, which fully demonstrated that endogenous BDNF had a better effect on promoting nerve repair than that of exogenous BDNF.

Finally, a small quantity of polyaniline particles was found in the regenerated nerve, it is different from our previous results of HSPS sponge subcutaneous implantation *in vivo* (Wu et al., 2019). When the HSPS sponges were implanted subcutaneous after 3 weeks, polyaniline particles had been cleared by the body tissues (Wu et al., 2019). Since the HSPS conduit is a lumen structure but the sponge embedded under the skin is a lumpy structure, macrophages cannot easily enter the conduits and can’t clear the polyaniline very quickly. So the polyaniline particles cannot be cleared within 12 weeks. It has also been reported that the size of polyaniline particles formed by the degradation of PLLA/polyaniline/TSA fiber scaffolds *in vivo* ranged from 2.5 to 3.1 nm, while macrophages were able to devour foreign particles of 10 nm size (Yao et al., 2019). Moreover, the polyaniline nanoparticles could be swallowed by macrophages, and foreign particles were mainly dispersed in vesicles and mitochondria of macrophages (Wang et al., 2019). In contrast to our results, the polyaniline nanoparticles produced after HSPS degradation did not cause inflammation in the heart, liver, spleen, lung, and kidney organs, and our results showed that polyaniline particles did not move to the organs. Therefore, HSPS conduits are safe when they are used *in vivo*.

## Conclusion

In this work, a kind of conductive HSPS sponge conduit was developed as conductive neural tissue engineering scaffold, and it was used to repair nerve defects with or without ES subsequently. The HSPS conduit under ES could facilitate animal nerve function and structure recovery, and upregulate the BDNF expression. Furthermore, the BDNF protein can promote Schwann cells proliferation and MBP expression. Therefore, the combination of HSPS conduit and ES in this work may have potential application in nerve tissue engineering field.

## Data Availability Statement

The raw data supporting the conclusions of this article will be made available by the authors, without undue reservation.

## Ethics Statement

The animal study was reviewed and approved by the Review Board of Hubei Medical Laboratory Animal Center.

## Author Contributions

PW and YZ conceived the initial idea and designed the experiments. PW, FC, AX, QDu, QDo, MK, and XL performed the experiments. PW, YZ, and FC analyzed the data and wrote the manuscript. QZ and YC guided the work and revised the manuscript. YC worked on funding acquisition. All authors have read and approved the final manuscript.

## Conflict of Interest

The authors declare that the research was conducted in the absence of any commercial or financial relationships that could be construed as a potential conflict of interest.
